# Optimizing adherence in HIV prevention product trials: Development and psychometric evaluation of simple tools for screening and adherence counseling

**DOI:** 10.1371/journal.pone.0195499

**Published:** 2018-04-12

**Authors:** Elizabeth E. Tolley, Kate Morrow Guthrie, Seth Zissette, Joseph L. Fava, Katherine Gill, Cheryl E. Louw, Philip Kotze, Krishnaveni Reddy, Kathleen MacQueen

**Affiliations:** 1 FHI 360, Durham, North Carolina, United States of America; 2 Centers for Behavioral & Preventive Medicine, The Miriam Hospital, Providence, Rhode Island, United States of America; 3 Deptartment of Psychiatry & Human Behavior, Alpert Medical School of Brown University, Providence, Rhode Island, United States of America; 4 Desmond Tutu HIV Centre, Cape Town, South Africa; 5 Madibeng Centre for Research, Brits, South Africa; 6 University of Pretoria, Department of Family Medicine, Faculty of Health Sciences, Hatfield, South Africa; 7 Qhakaza Mbokodo Research Clinic, Ladysmith, South Africa; 8 Wits Reproductive Health & HIV Institute, Johannesburg, South Africa; University of Toronto Dalla Lana School of Public Health, CANADA

## Abstract

**Background:**

Low adherence in recent HIV prevention clinical trials highlights the need to better understand, measure, and support product use within clinical trials. Conventional self-reported adherence instruments within HIV prevention trials, often relying on single-item questions, have proven ineffective. While objective adherence measures are desirable, none currently exist that apply to both active and placebo arms. Scales are composed of multiple items in the form of questions or statements that, when combined, measure a more complex construct that may not be directly observable. When psychometrically validated, such measures may better assess the multiple factors contributing to adherence/non-adherence. This study aimed to develop and psychometrically evaluate tools to screen and monitor trial participants’ adherence to HIV prevention products within the context of clinical trial research.

**Methods and findings:**

Based on an extensive literature review and conceptual framework, we identified and refined 86 items assessing potential predictors of adherence and 48 items assessing adherence experience. A structured survey, including adherence items and other variables, was administered to former ASPIRE and Ring Study participants and similar non-trial participants (n = 709). We conducted exploratory factor analyses (EFA) to identify a reduced set of constructs and items that could be used at screening to predict potential adherence, and at follow-up to monitor and intervene on adherence. We examined associations with other variables to assess content and construct validity. The EFA of screener items resulted in a 6-factor solution with acceptable to very good internal reliability (α: .62-.84). Similar to our conceptual framework, factors represent trial-related commitment (Distrust of Research and Commitment to Research); alignment with trial requirements (Visit Adherence and Trial Incompatibility); Belief in Trial Benefits and Partner Disclosure. The EFA on monitoring items resulted in 4 Product-specific factors that represent Vaginal Ring Doubts, Vaginal Ring Benefits, Ring Removal, and Side Effects with good to very good internal reliability (α = .71-.82). Evidence of content and construct validity was found; relationship to social desirability bias was examined.

**Conclusions:**

These scales are easy and inexpensive to administer, available in several languages, and are applicable regardless of randomization. Once validated prospectively, they could (1) screen for propensity to adhere, (2) target adherence support/counselling, and (3) complement biomarker measures in determining true efficacy of the experimental product.

## Introduction

The prophylactic use of antiretroviral drugs (ARV) to prevent HIV transmission has proven moderately to highly efficacious in several clinical trials testing the use of a peri-coital vaginal gel [1], daily oral pills [2–4], and, most recently, vaginal rings [5]. Despite these successes, adherence–both its measurement and optimization–has emerged as a serious challenge for HIV prevention clinical trial research. In two large trials of heterosexual African women (the Vaginal and Oral Interventions to Control the Epidemic or VOICE trial and the FEM-PrEP trial) [6–8], inability to determine product effectiveness was attributed to poor adherence to daily vaginal (VOICE) and oral (VOICE and FEM-PrEP) dosing regimens, based on low or no drug detection in plasma from random subsamples of women in the active study arms. Furthermore, even in trials that have shown product efficacy, adherence has been highly variable. For example, adherence levels lower than 50% led investigators to discontinue recruitment in two sites in the ASPIRE trial [5]. In iPrEx, adherence varied regionally, with highest objectively measured adherence in MSM in sites in the United States and lowest levels in Peru [9]. Overall, adherence was high in the Partners PrEP trial among HIV discordant couples with known risk for HIV, but low in other prevention trials of African women. In several trials, younger women had higher HIV incidence and lower adherence than older women [5, 8].

Several qualitative sub-studies further examined women’s experiences with product adherence and reasons for non-use. In Fem-PrEP, participants classified as highly adherent tended to describe their commitment to trial goals for either altruistic reasons or because they believed that trial participation would reduce their own risk; they developed habits or relied on tools to ensure they adhered to product use and trial visits and reported having support from partners and other community members [10]. Partner and community concerns about the safety and potency of the drugs being tested in the VOICE trial may have reduced women’s motivation to adhere to product use [11]. When asked post-trial about potential reasons for non-adherence, some former VOICE participants acknowledged their own occasional non-use of study product due to forgetfulness or boredom, but almost all suggested that intentional non-use was common among other trial participants, due to their own or others’ distrust of the trial and/or of the product [12]. Indeed, among a sample of former VOICE participants randomized to an active product and for whom pharmacokinetic results existed, at least 50% had no evidence of tenofovir in their blood samples [8, 13]. Women in this sub-study suggested that they would be more likely to adhere to product use, if they knew they could be objectively monitored [13].

Because high and sustained adherence is essential to the evaluation of product safety and efficacy in clinical trials [14], there is an urgent need to develop objective biomarkers of adherence [15–17]. Nevertheless, the unique aspects of clinical trial participation may continue to attract some participants who have little incentive to adhere (or strong incentive to adhere only before visits)–or who face barriers to adherence that emerge primarily from the clinical trial context itself [18, 19]. Furthermore, while objective measures can indicate suboptimal adherence, they cannot identify reasons for, or guide approaches to intervening on, non-adherence. For these reasons, additional approaches to measuring and optimizing adherence should also be considered [20].

An approach which has long been used to assess treatment non-compliance [21–23], involves summated rating scales, which are composed of multiple items in the form of questions or statements that, when combined, measure a more complex construct that may not be directly observable [24, 25]. Validated multi-item measures tend to be more stable, reliable and precise than single items because they produce a set of internally consistent replies that are “less prone to socio-psychological biases”, enabling the random error in the measure to be minimized or canceled out [26]. As such, they may better assess the multiple factors likely to contribute to ring non-adherence.

### Project overview

The overall goal of the project was to develop and provide initial psychometric validation for a set of scale measures that may prove inexpensive and easy to administer, with better predictive ability than current self-report adherence measures. Two types of scales were envisioned: 1) Screener measures, administered prior to product use (i.e., during a screening visit) that might predict an individual’s propensity to adhere; and 2) Adherence Monitoring measures that could be administered periodically during participant follow-up to assess participants’ attitudes about and experiences with the product.

We followed a standard scale development process, which included identifying an item and construct pool from the published literature related to adherence measurement and HIV prevention (specifically microbicide) clinical trial research; rounds of cognitive interviewing to improve item framing, comprehensibility and salience; and administration of a survey to facilitate exploratory factor analyses and psychometric evaluation of resulting constructs. After this initial evaluation, we envisioned sharing the resulting scale measures with other clinical researchers, who could prospectively evaluate the scales’ ability to predict adherence among participants in a new HIV prevention clinical trial.

### Conceptual framework

We conducted a comprehensive review of the peer-reviewed literature to identify articles describing psychometrically validated scales or other measures related to adherence or compliance. We included measures of adherence in the context of both treatment and prevention to a range of conditions, including HIV, pregnancy prevention, and other disease areas (i.e., blood pressure, diabetes). Measures assessing adherence within clinical trial contexts were of particular interest. In addition, we conducted additional searches on measures assessing motivations for clinical trial participation, as well as measures of social desirability bias.

**Fig 1:**
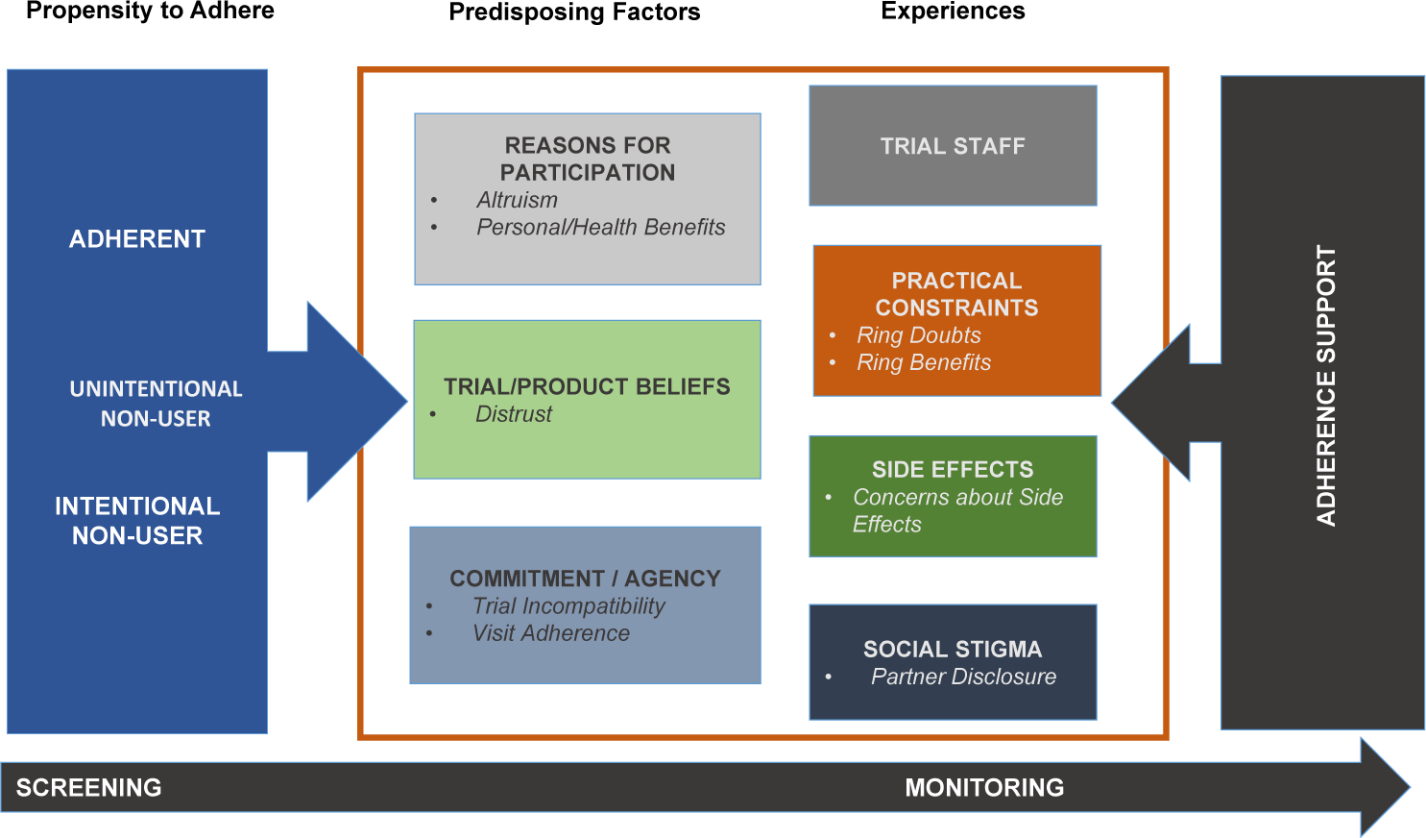
Conceptual framework.

Based on the literature review, we developed a conceptual framework (Fig 1) depicting potential domains for adherence screening and monitoring tools. As depicted on the left-hand side of the diagram, our screener tool would ideally identify whether a potential trial participant was likely to be adherent, unintentionally non-adherent, or intentionally non-adherent. These adherence categories are based in the Necessities-Concerns Framework [27], a conceptual model postulating that adherence to a range of chronic disease medications is explained by an individual’s perceived need for treatment and their concerns about the potential consequences of taking it [28]. Despite limited application of the framework to prevention, findings from the qualitative studies described earlier are consistent with the framework’s adherence constructs. Predisposing factors which emerged in the qualitative HIV prevention literature and for which we identified potential screening measures include: reasons for trial participation [29–36]; beliefs about clinical trials or the products being tested [37, 38]; and personal attributes such as commitment, self-efficacy, or agency [21, 38–40]. The literature review also identified barriers and facilitators of adherence that might be experienced once a trial participant began using the study product and which could be assessed over time, including: the beneficial or negative impact of trial staff [38, 41, 42]; practical constraints, including travel, cost of participation, and impact on daily life [22, 37, 43, 44]; experience of side effects [44–46]; and impact of stigma [30, 44]. These constructs are presented on the right-hand side of our conceptual framework. Finally, the larger box within which predisposing and experienced factors are located represents the potential for some participants to respond in a socially desirable manner. We reviewed and included measures of social desirability bias in this study [47–50]. In this paper, we describe the outcomes of our survey and initial validation procedures.

## Methods

Between February and June 2016, we administered a cross-sectional survey in four South African clinical trial sites located in Johannesburg, Cape Town, Madibeng and Ladysmith. Prior to implementation, the study was reviewed and approved by the FHI 360 Protection of Human Subjects Committee, the Wits Human Research Ethics Committee in Johannesburg, and Pharma Ethics for Madibeng and Ladysmith sites. The survey was administered electronically on tablets by same-sex interviewers, with a total of 709 women. Eligibility included being between the ages of 18–40 and willingness to provide written informed consent which was captured electronically, prior to taking part in the survey. Participants were both former participants (FTP) of vaginal ring or other HIV prevention clinical trials and trial-naïve participants (TNP). Participants could choose to hear and respond to all or individual survey items in English or their local language (i.e., isiZulu in Johannesburg and Ladysmith, Xhosa in Cape Town, and Tswana in Madibeng.)

The survey was divided into three sections. All women regardless of trial experience responded to 76 “screener” items measuring potential predictors of adherence; 26 culturally relevant items from the Marlowe-Crowne social desirability scale (SDB) [49] were also distributed in groups within this section. Participants received a point for each item in which their response was in the more socially-desirable direction. Only former trial participants responded to an additional set of 48 monitoring items assessing product-specific adherence attitudes and behaviors. A six-point scale ranging from “1 = Disagree A Lot” to “6 = Agree A Lot” was used for potential adherence screening and monitoring items. The last section, administered to all participants, included additional demographic and psychosocial variables, including age and education level, as well as whether a participant earned her own income (0 = no; 1 = yes), believed her partner had other partners (0 = no; 1 = unsure; 2 = yes) and whether she was 1 = willing, 2 = persuaded, or 3 = forced to have sex at the last sex act.

### Exploratory dimensional analysis (EDA)

We conducted separate EDAs of screening and monitoring items. Prior to each EDA, we first examined the response distribution, means, standard deviations, skew and kurtosis of all items, eliminating items with very high mean scores (> 5.8 of maximum 6.0). With each set of items, we conducted an exploratory factor analysis (EFA) with oblique (Promax) rotations and a principal component analysis (PCA) with orthogonal (varimax) rotation, retaining items with loadings of .4 or higher on both procedures, and assessing similarities in the resulting factor structure of the two analyses.

We used both the scree test [51] and the parallel analysis (PA) procedure [52, 53] in our initial determination of the underlying dimensional structure of the screening and the adherence monitoring measures. We also examined the internal consistency of each derived factor and used Cronbach’s Coefficient Alpha statistic [54] to help refine an individual scale by removing items with low inter-item correlations to increase reliability. Our final decision as to whether to keep or remove a specific item was based on the conceptual content of each item as it related to a specific factor.

### Initial validation

We qualitatively evaluated the fit between our conceptual framework and the resulting constructs. In addition, we made predictions about the direction and strength of correlations between our adherence screener and monitoring constructs, as well as between scale constructs and other sociodemographic variables. We ran Pearson Product Moment Correlations to test our predictions. Finally, we reran our EDA of screener items separately for FTP and TNP samples to assess whether a different factor structure and/or item composition existed in these two groups.

## Results

### Sociodemographic information

Overall, 60% of survey participants (n = 425) had participated in past HIV prevention clinical trial research; the majority (n = 339: 79.8%) were former participants of ASPIRE or the Ring Study–two trials evaluating the efficacy of an HIV prevention intravaginal ring, while 74 (17.4%) women had used a vaginal gel in the FACTS trial and 12 (2.8%) had used oral pills in the VOICE trial (Table 1). Trial-naïve participants, who were drawn from the same communities, tended to be younger, but similar to the FTP group on other sociodemographic information.

**Table 1:** Sociodemographic characteristics.

	All (n = 709)	FTP^1^ (n = 425)	TNP^2^ (n = 284)	P-Value
Age (mean, SD)	28.5, 6.4	29.7, 6.1	26.8, 6.4	<0.001***
**Education**:	%	%	%	0.336
< Secondary	35	38	33	
Secondary	43	43	44	
> Secondary	22	19	23	
**Past trial participation**:	%	%	n/a	n/a
	60	100		
Vaginal ring	48	80		
Vaginal gel	10	17		
Oral pill	2	3		
**Lives in**:	%	%	%	0.039*
House or townhouse	46	50	41	
Flat or single room	14	15	14	
Shack	23	20	27	
Other	16	15	18	
Earns own income	%	%	%	<0.001***
	50	42	56	
**Relationship**:	%	%	%	0.560
Married, living as married	24	25	22	
Regular partner, not living together	67	67	67	
Sexually active, no partner	3	2	4	
Currently not sexually active	6	6	7	
Years with current partner (mean, SD)	(n = 643) 6.5, 5.4	(n = 389) 7.1, 5.5	(n = 254) 5.5, 5.1	<0.001***
Age difference of main sex partner (mean, SD)	(n = 643) 4.1, 3.8	(n = 389) 4.1, 3.7	(n = 254) 4.1, 3.9	0.832
Partner provides financial/material support	%	%	%	0.01**
	87	90	83	
**Partner has other partners**:	%	%	%	0.252
Unsure	59	61	55	
Yes	14	13	15	
**Willingness to have sex at last encounter**:	%	%	%	0.274
Willing or wanted to have sex	97	98	96	
Persuaded or coerced	2	2	3	
Physically forced or raped	1	1	1	
Used condom at last sex encounter	%	%	%	0.485
	59	60	58	
**Social Desirability Bias Score** (mean, SD)	18.1, 3.1	18.3, 3.2	17.8, 2.9	0.023*
% Low (1 SD below mean)	14	13	15	
% Average	73	72	75	
% High (1 SD above mean)	13	15	10	

^1^FTP = Former Trial Participant

^2^TNP = Trial-Naïve Participant

*p-value < .05

**p-value < .01

***p-value < .001

### Screener results

We began with 86 potential screening items and retained 40 items, loading onto six factors (Table 2). The 6-factor solution explained 64% of the variance in the full sample and 57% and 48% in the FTP and TNP samples respectively. Internal reliability of items ranged from acceptable for the Commitment to Research factor (α = .61) to very good for the Partner Disclosure factor (α = .80).

**Table 2:** PCA item loadings for 6-factor solution of screening items, from the full sample and by sub-groups.

SCREENING TOOL	FULL	FTP	TNP
*COMMITMENT TO RESEARCH*	8 items *a = .61*	8 items *a = .58*	7 items *a = .60*
1. I want to participate in HIV prevention research because I am proud to help in the fight against AIDS.	0.51	0.45	0.70
2. I want to participate in HIV prevention research because I want to contribute to scientific information.	0.50	0.46	0.51
3. I want to participate in HIV prevention research because finding new HIV prevention products would be worth any inconvenience of participating.	0.50	0.45	n/a
4. I want to participate in HIV prevention research because I want to help the researchers.	0.48	0.49	0.51
5. I want to participate in HIV prevention research because I feel my participation in research will help find effective HIV prevention products.	0.48	0.58	n/a
6. The idea of participating in research is appealing to me.	0.45	0.55	n/a
7. I have made a commitment to use the research product as instructed.	0.45	0.49	n/a
8. I want to participate in HIV prevention research because I feel that I will benefit from the research, whether the product works or not.	0.41	0.43	0.41
9. My friends and family know that I am participating in research.	n/a	n/a	0.54
10. I am fully confident I can use the research product even if I experience strong side effects.	n/a	n/a	0.44
11. I am not interested in activities that will expand my experiences. *(reverse scored)*	n/a	n/a	-0.42
*PERSONAL & HEALTH BENEFITS*	5 items *a = .69*	7 items *a = .60*	6 items *a = .66*
1. I want to participate in HIV prevention research because I am motivated by free medical check-ups.	0.68	0.68	0.70
2. I want to participate in HIV prevention research because I want free health care.	0.67	0.66	0.58
3. I want to participate in HIV prevention research because I think the research will improve my health.	0.57	0.51	n/a
4. I want to participate in HIV prevention research because I want to reduce my chances of getting HIV.	0.50	0.49	n/a
5. I want to participate in HIV prevention research because I want to obtain the latest HIV information.	0.43	n/a	0.41
6. I believe that people who participate in HIV prevention research can experience unpleasant side effects.	n/a	-0.45	n/a
7. I do not care whether my family knows about my involvement in the research.	n/a	0.50	n/a
8. I believe that the staff will give me a product that works.	n/a	0.40	n/a
9.The idea of participating in research is appealing to me.	n/a	n/a	0.52
10. I might decide to participate in the research because I’m curious.	n/a	n/a	0.52
11. I want to participate in HIV prevention research because I want to be valuable to the community.	n/a	n/a	0.50
*DISTRUST of RESEARCH*	11 items *a = .78*	10 items *a = .77*	11 items *a = .77*
1. I do not trust research in general.	0.66	0.64	0.65
2. I might lose more than I gain by participating in HIV prevention research.	0.58	0.55	0.59
3. Researchers at the clinic are not truthful.	0.56	0.54	0.55
4. I admit I am distrustful of foreign (white) HIV prevention researchers.	0.55	0.51	0.56
5. I worry that participating in research could lead to future health problems.	0.55	0.49	0.55
6. I do not want to be used for an experiment.	0.54	0.54	n/a
7. It's hard to believe that the research product will help me.	0.53	0.58	0.47
8. I am worried that the product is experimental.	0.51	0.57	n/a
9. I do not trust that the research product can prevent HIV.	0.47	0.52	0.42
10. I am worried that I will get HIV because I am using the research product.	0.46	n/a	0.65
11. The research will take a lot of my time.	0.43	0.50	n/a
12. I am concerned that people may harass me for participating in this research.	n/a	n/a	0.45
13. I was not given enough time to decide if I wanted to participate in the research.	n/a	n/a	0.43
14. I believe that people who participate in HIV prevention research can experience unpleasant side effects.	n/a	n/a	0.47
*TRIAL INCOMPATIBILITY*	8 items a = .69	8 items a = .69	10 items a = .71
1. I want to participate in HIV prevention research because I am motivated by the money I get for participating in the research.	0.56	0.54	0.47
2. People who participate in HIV prevention research may be rejected by others.	0.55	0.61	n/a
3. I like taking risks. *(reverse scored)*	-0.54	-0.63	n/a
4. I often change my mind about decisions if my friends and family disagree.	0.50	0.46	0.45
5. I think the care I get as a research participant is the same as the care I get in the local clinic.	0.48	0.48	0.49
6. I am fully confident I can use the research product even if I experience strong side effects.	0.47	0.47	n/a
7. I am concerned that people may harass me for participating in this research.	0.44	0.51	n/a
8. Luck plays a big part in determining my health.	0.43	n/a	0.68
9.I think I will like using the research product more than condoms.	n/a	0.45	n/a
10. On a few occasions, I have given up doing something because I didn’t think I could do it.	n/a	n/a	0.55
11. God plays a big part in determining how my health is.	n/a	n/a	0.52
12. The type of help I receive from other people determines how well I do in the research.	n/a	n/a	0.51
13. The research will take a lot of my time.	n/a	n/a	0.50
14. I have difficulty arranging my life in a way that is satisfying to me.	n/a	n/a	0.42
15. It is sometimes hard for me to finish things if I am not encouraged.	n/a	n/a	0.42
*PARTNER DISCLOSURE*	5 items *a = .80*	5 items *a = .83*	5 items *a = .77*
1. It is important for my partner to know that I am participating in research.	0.84	0.86	0.80
2. I will tell my partner about my involvement in the research.	0.84	0.87	0.77
3. My partner knows that I am participating in the research.	0.78	0.87	0.57
4. I would not tell my partner that I am participating in research.	-0.69	-0.73	-0.63
5. Letting my partner know about my participation is “fear”.	-0.47	-0.49	-0.45
*VISIT ADHERENCE*	3 items*a = .67*	3 items *a = .68*	3 items *a = .64*
1. I have never been late for an appointment.	0.62	0.59	0.60
2. I never miss an appointment.	0.57	0.52	0.56
3. I always do what the doctor tells me.	0.57	0.62	0.51

The 6-factor solution mapped well to our conceptual framework (Fig 1), consisting of factors that measured reasons for clinical trial participation (Commitment to Research, Personal & Health Benefits), beliefs about the trial and product (Distrust of Research) and commitment/agency (Trial Incompatibility, Partner Disclosure, Visit Adherence). While the full and subgroup EDAs produced similar underlying factor structures, item composition differed for several factors. For example, in the TNP subgroup, the Distrust factor did not include two items related to the use of experimental products, however, the TNP Distrust factor did include the item “*I believe that people who participate in HIV prevention research can experience unpleasant side effects*.*”* This same item did not load on Distrust for the FTP subgroup, but instead, loaded negatively onto the Personal & Health Benefits factor. Additionally, several items indicating potential risk disinhibition (i.e., belief in the therapeutic benefit of an experimental product) loaded onto the Personal & Health Benefits factor for the FTP subgroup, but did not do so for the TNP subgroup. The Trial Incompatibility factor showed the largest differences in item composition by subgroup. Over the full sample, the items suggest participants’ incompatibility or non-alignment with the trial, including being motivated by study reimbursements and having concerns about how others would react to their trial participation. Within the TNP subgroup, most items loading on this factor suggested an external locus of control–that God, luck or others determined their ability to accomplish behaviors. Many of these items did not load onto the Trial Incompatibility factor in the FTP subgroup analysis.

### Adherence monitoring results

Adherence monitoring items were adapted during our cognitive interviewing process (not described) to assess attitudes and experiences specific to product use. Because our survey included very few vaginal gel or pill users, we conducted this analysis with FTPs of previous vaginal ring trials only (n = 339). Out of 48 potential adherence monitoring items, we retained 29 items loading on four factors (Table 3), including an “enacted” adherence measure (Ring Removal), several factors related to the presence or absences of practical constraints (Vaginal Ring Doubts and Vaginal Ring Benefits) and Side Effects. The 4-factor solution explained 64% of the variance in items. The resulting scales had moderate to high internal reliability. Although no specific factors emerged that related to Trial Staff or Social Stigma, several items from these domains did load onto the Vaginal Ring Doubts factor.

**Table 3:** EFA item loadings for 4-factor solution of adherence monitoring items, FTP sample from vaginal ring clinical trials only.

MONITORING TOOL	FTP
*Vaginal Ring Removal*	7 items *a = .82*
1. There were times when I removed and reinserted the vaginal ring.	0.74
2. If I took out my vaginal ring to have sex, I sometimes forgot to put it back in for several hours.	0.74
3. I did not remove the vaginal ring at any time, except during a clinic visit. *(reverse scored)*	-0.73
4. I removed the vaginal ring when I had sex with my partner.	0.72
5. I removed the vaginal ring during my menses.	0.69
6. I sometimes removed the vaginal ring to clean it.	0.54
7. Sometimes, if I felt worse when I had the vaginal ring in my body, I stopped using it.	0.46
*Vaginal Ring Doubts*	8 items *a = .78*
1. I was tempted to stop using my vaginal ring when side effects began to interfere with daily activities.	0.81
2.The effects of the vaginal ring lasted even if I removed it for several hours.	0.76
3. I often thought the research staff didn’t tell me everything they know about the research.	0.65
4. Using the vaginal ring some of the time is better than not using it at all.	0.62
5. It was hard to believe that using the vaginal ring would help me.	0.50
6. I had doubts about the benefits of using the vaginal ring.	0.49
7. I was not always sure that I inserted the vaginal ring correctly.	0.42
8. It was difficult for me to explain the research to my friends.	0.40
*Vaginal Ring Benefits*	9 items *a = .75*
1. Using the vaginal ring improved my outlook on life.	0.67
2. Wearing the vaginal ring gave me confidence.	0.67
3. The vaginal ring helped me to feel better physically.	0.66
4. I believed that my risk for HIV infection was the same whether I used the vaginal ring or not. *(reverse scored)*	-0.54
5. The vaginal ring improved my sex life.	0.52
6. I believed that my risk for HIV infection was less when I was using the vaginal ring.	0.48
7. I believed that the vaginal ring would reduce my chance of getting HIV.	0.46
8. The vaginal ring worked as soon as I inserted it.	0.44
9. I believed that I might get HIV if I don't use the vaginal ring as instructed.	0.43
*Concern about Side Effects*	5 items *a = .61*
1. Side effects made it difficult for me to keep using the vaginal ring.	0.65
2. Vaginal ring side effects interfered with my everyday life.	0.63
3. There was a chance that the vaginal ring might cause me harm.	0.58
4. Vaginal ring side effects interfered with my sex life.	0.50
5. I was sometimes afraid the ring would get lost in my body.	0.43

### Social desirability

Overall, trial-naïve participants scored lower on the social desirability measure than former trial participants. Participants who scored high (1 SD above the mean) on the SDB scale scored significantly lower on the Distrust in Research and Trial Incompatibility screening scales as well as the Vaginal Ring Doubts and Concern about Side Effects monitoring scales; they also scored significantly higher in the Commitment to Research, Visit Adherence and Vaginal Ring Benefits scales (Table 4).

**Table 4:** Summary of scale scores by participant type and SDB levels.

Scale	Mean			S. Dev.	IQR	Mean by SDB Level		
*Screening*	FTP	NTP	All	All	All	Low	Mid	High
Distrust	2.06	2.26	2.14	0.92	1.28	2.49*	2.16	1.71*
Trial Incompatibility	3.30	3.51	3.38	0.96	1.44	3.44	3.42	3.09*
Personal & Health Benefits	4.81	4.93	4.86	0.58	0.62	4.86	4.86	4.90
Partner Disclosure	4.00	3.77	3.91	0.71	0.20	3.83	3.94	3.83
Visit Adherence	4.32	4.42	4.36	0.90	1.20	3.91*	4.41	4.52*
Commitment to Research	5.69	5.63	5.67	0.46	0.56	5.55	5.67	5.76*
*Monitoring*								
Vaginal Ring Removal	1.97		1.97	0.60	0.15	2.13	1.97	1.85
Vaginal Ring Doubts	2.43		2.43	1.25	1.50	2.73	2.46	2.01*
Vaginal Ring Benefits	4.67		4.67	0.77	1.00	4.32*	4.68	4.90*
Concerns About Side Effects	1.69		1.69	0.90	1.00	2.16*	1.67	1.33*

*p-value < .05

**p-value < .01

***p-value < .001

### Initial construct validation

After developing the scales, we made *a priori* predictions about the correlations between screener constructs and several sociodemographic variables, as well as with the adherence follow-up measures (Table 5). The bolded cells in Table 4 show where our predictions matched findings. Few of our screener constructs performed as hypothesized relative to the sociodemographic variables. In many cases, we assumed significant relationships between screening scale scores and demographic information when correlations were not significant. However, our predictions of correlations between screener and adherence monitoring scales were often correct. In particular, participants who scored higher on Distrust in Research–and lower on Visit Adherence–were more likely to have high scores on Vaginal Ring Removal, Vaginal Ring Doubts and Side Effects. Conversely, high scores on Personal & Health Benefits and Visit Adherence were associated with high scores on Vaginal Ring Benefits. The Partner Disclosure scale was negatively correlated with Vaginal Ring Removal, but not associated with other monitoring scales. As predicted, participants with low scores on our SDB measure had higher scores on Distrust in Research, as well as Vaginal Ring Removal, Vaginal Ring Doubts and Side Effects.

**Table 5:** Predictions of correlations between screener scores, concurrent variables and adherence monitoring scores.

		Age	Educa-tion	Own income	Partner’s partners	Forced sex	VR Removal	VR Doubts	VR Benefits	Side Effects	SDB
		*Socio-demographic Variables*					*Monitoring Scales*				
*Screening*	Commitment to Research	**NA**	Pos	Pos	Neg	**NA**	Neg	Neg	Pos	NA	**Pos**
		**0.05**	0.00	0.02	-0.02	**-0.02**	-0.04	-0.04	0.04	-0.16*	0.12*
	Distrust in Research	**NA**	Neg	Neg	Neg	NA	**Pos**	**Pos**	**Neg**	**Pos**	**Neg**
		**-0.09**	0.03	0.01	0.12*	0.13*	0.21*	0.42*	-0.36*	0.53*	-0.31*
	Personal/Health Benefits	NA	Pos	Neg	**Pos**	**NA**	Neg	Neg	**Pos**	Neg	NA
		-.12*	-0.08	0.22*	0.22*	**0.06**	0.03	0.05	0.30*	0.02	-0.25*
	Trial Incom-patibility	Neg	**Neg**	**NA**	Pos	NA	Pos	**Pos**	**Pos**	**NA**	Neg
		0.14*	-0.13*	**-0.07**	-0.18*	-0.24*	0.09	0.67*	0.13*	**0.09**	0.34*
	Partner Disclosure	Pos	Pos	**NA**	Neg	Neg	**Neg**	NA	**NA**	**NA**	NA
		-0.03	-0.08	**0.02**	-0.01	-0.03	-0.12*	-0.16*	**0.09**	**-0.05**	0.11*
	Visit Adherence	**NA**	Pos	**NA**	NA	Neg	**Neg**	**Neg**	**Pos**	NA	**Pos**
		**0.04**	-0.03	**-0.04**	-0.15*	0.06	-0.25*	-0.35*	0.37*	-0.17*	0.08*
	SDB	NA	**NA**	**NA**	NA	**NA**	**Neg**	**Neg**	**Pos**	**Neg**	
		0.18*	**-0.05**	**-0.03**	-0.12*	**-0.05**	-0.14*	-0.22*	0.22*	-0.30*	

*p-value < .05

**p-value < .01

***p-value < .001

Correlations between Screening scales and sociodemographic and behavioral variables (as identified in Table 1) shown in non-shaded cells. Correlations between Screening and Monitoring scales shown in shaded cells. Bolded text represents confirmation of hypothesized relationship.

## Discussion

The inability of several recently concluded trials to determine the efficacy of new HIV prevention products due in part to low product adherence has prompted the field to consider alternative strategies for trial implementation [55]. This study was based on the premise that achieving product adherence within randomized, placebo-controlled HIV prevention trials is complex, requiring participants to possess not only accurate knowledge and behavioral skills, but also sufficient motivation, social support and life organization. If it were possible to identify and either screen out or more effectively monitor and support individuals with a low propensity to adhere to product use, the ability of a clinical trial to determine product efficacy would be increased. Our study developed two sets of tools, one set to identify individuals at screening who exhibit substantial adherence-related challenges and a second set aimed at monitoring adherence challenges over the course of trial participation with the goal of achieving better overall adherence during the trial. All scale items have been thoroughly evaluated in English and three South African languages for comprehensibility, salience and ease of response. Although not presented in this paper, product-related items for monitoring scales have also been adapted to other product contexts, including oral pills and vaginal gels.

### Screening measures

This study produced a set of six scale measures that have the potential to identify individuals at screening who may face challenges with adherence to clinical trial requirements and product use. Two of the screening scales, Commitment to Research and Personal & Health Benefits, represent reasons identified by others as underlying motivations to participate in a range of prevention trials [36, 56–59]. Altruism (a component of the Commitment to Research scale), expressing feelings or actions that show a selfless concern for others, was identified as an independent predictor of adherence in a randomized, placebo controlled trial assessing use of estrogen for the prevention of stroke [60]. It was endorsed by the vast majority of participants in the VaxGen trial as a primary reason for joining the trial [31, 61]; it was also identified in qualitative sub-studies with men-who-have-sex with men (MSM) from U.S. sites of the iPrEX trial [62]. While women participating in the ADAPT open-label pre-exposure prophylaxis (PrEP) study in Cape Town, South Africa were engaged in research to benefit themselves, many also identified “Ubuntu”–contributing to the wellbeing of one’s community–as a strong motivator for study participation [63]. Women, selected to participate in a Fem-PrEP sub-study whose mean drug concentration levels throughout the trial were moderate to high, reported that a mix of personal benefits and altruism motivated their adherence to study product [10]. Indeed, personal benefits, such as access to good healthcare, access to social services or routine testing for HIV and other conditions, were more frequently mentioned as a reason to participate in HIV vaccine research among high-risk drug-using women in the U.S. [64], as well as women participating in a PrEP trial in South Africa [65].

A third set of items produced from our screening items related to Distrust of Research, a construct identified as limiting trial participation among African Americans in cardiovascular trials [66] and HIV prevention trials [36, 67, 68], high-risk women [64] and minorities in the U.S.[68]. In the VOICE-C sub-study, both women and men in Uganda, Zimbabwe and South Africa described how a lack of understanding and trust in research and the product being tested made it difficult for some women to adhere to product use [69]. Similarly, South African women participating in the qualitative sub-study of the ADAPT open-label PrEP study revealed an underlying level of skepticism about PrEP and HIV prevention research more generally [63]. As the authors note, this skepticism did not lead women to reject the research outright, but did lead some to approach study and product use requirements cautiously. Based on their qualitative data, they theorize that participants who hold strong feelings of fear and distrust may still join PrEP trials or open-label studies, particularly if they see high value from other aspects of trial participation, while intentionally avoiding PrEP use and actively discouraging others from using their products.

Additionally, three other potential screening scales assess aspects of the participants’ personal, social and daily contexts that affect their ability to adhere to trial and product use. They include a 15-item Trial Incompatibility scale, a five-item Partner Disclosure scale, and a three-item Visit Adherence scale. In contrast to the Distrust in Research scale, the Trial Incompatibility scale appears to represent attitudes towards health and a life context that is disorganized and therefore doesn’t support the exigencies of clinical trial participation. Although the composition of scale items substantially differs between former trial participants and those who were trial-naïve, they both include items indicating motivation for monetary rather than health reasons for trial participation and that others may influence their health and/or trial-related behaviors. Both also have items suggesting incompatibility with the trial due to disliking risk or the time commitment required by participation. The scale solution produced from the trial-naïve sample also includes several items suggesting that other forces outside of their control (God or fate, for example) influence their behaviors. The Perceptions and Practicalities Approach, a framework developed to explain non-adherence of therapeutic medications, suggests that unintentional non-adherence is driven by lack of capacity and resources which might include perceived difficulty in organizing their life circumstances, lack of social support [28, 70]. The inability to disclose to sexual partners about one’s participation in research and use of study products has been identified by numerous researchers as barrier to product use [13, 71]. The Visit Adherence scale may tap into an individual’s commitment and/or ability to organize their daily lives around health visits, a construct that has been shown to relate to adherence [72].

### Monitoring scales

We produced a set of four monitoring scale measures that could be used to tailor counseling and address specific barriers or concerns to product use over trial follow-up. The Vaginal Ring Removal scale is composed of items expressing circumstances in which participants may have removed their ring since the previous follow-up visit. Because participants can express a degree of (dis)agreement to the items, they may be more willing to indicate problems with adherence than if asked direct “yes” or “no” questions. The Vaginal Ring Doubts, Vaginal Ring Benefits and Side Effects scales could provide counselors with some indications of whether ring use is perceived as potentially harmful or beneficial and how well it fits into their daily lives and sexual contexts. Several items on the Vaginal Ring Benefits scale indicated belief in the therapeutic benefit of ring use–a belief that might not be accurate within the context of a randomized, placebo-controlled trial.

### Social desirability bias

Ancillary studies from several past HIV prevention trials have found that a non-negligible group of women report high adherence despite having little or no intention of using their study product [7, 13]. However, to date, there do not appear to be any tools available to identify participants who are predisposed to exaggerate their adherence. We adapted and included a measure of social desirability bias, the Marlowe-Crowne scale, to explore, through cognitive interviewing, whether these items translated cross-culturally, and if so, whether the set of items might be used in combination with other scale scores to identify women with potential adherence problems. The Marlowe-Crowne scale has been described as assessing two underlying factors that lead individuals to misrepresent their behaviors–impression management and self-deception [50]. Because the SDB measure was associated in predictable ways with our screening and monitoring measures, it might be a useful tool to identify a subset of participants whose reported trial-related perceptions and behaviors are potentially “too good to be true”. However, before using such a tool to actively screen out potential participants, further research is needed to determine the predictive validity of the measure as it relates to an objective adherence measure. It is notable that SDB scores in our study were higher, on average, among former trial participants than women who had not previously participated in an HIV prevention trial. It is unclear, however, whether trial participation itself conditions individuals to manage the impressions that others have of them, whether those who choose to join trials differ from non-participants on one or both of these underlying factors (i.e., impression management and self-deception), or whether they are generally just more agreeable people.

We believe that the scale approach pursued in this study offers a novel and promising strategy to improving adherence within future HIV prevention clinical trials. Nevertheless, our study has several limitations. First, screening items could perform differently if individuals believe their responses could affect enrollment. Further research is needed to assess the predictive validity of our screening scales, as well as the timing and approach to their administration. We invite collaboration with clinical researchers to integrate these screening and/or monitoring scales into future trials or demonstration studies to determine how well one or more of these scales correlate with objective biomarkers of adherence. Second, the psychometric properties of several scales could be improved. For example, several screening scales, including Commitment to Research, Personal and Health Benefits and Partner Disclosure, are moderately to highly negatively skewed, meaning that a large proportion of participants reported high levels of agreement with items. Without prospective validation, it is difficult to determine whether this is problematic, reducing the scales’ ability to provide meaningful information about participants’ trial-related motivations and partner contexts. On the other hand, perhaps they are adequately sensitive and specific enough to identify the subset of participants whose low levels of commitment, concerns about health impacts, or inability to disclose to a partner would drive intentional non-adherence. Finally, the factor solutions for several of our scales differed for women who had previous trial-experience versus those who were trial naïve. Because most HIV prevention trials will recruit women with a range of trial experience, researchers may want to consider using all items initially, assessing item loading and internal reliability of each scale before analysis.

## Conclusion

Product adherence is essential for the successful evaluation of new HIV prevention candidates in clinical trials, but determining how best to measure and optimize adherence remains elusive. Increasingly, trials are relying on objective markers to detect whether products are being used–yet these measures provide only part of the picture. They do not, for example, provide information about why a participant has low or no evidence of product use, or whether and how such adherence barriers can be resolved. Therefore, despite shortcomings, behavioral adherence assessments which are inexpensive, non-invasive, and allow for immediate feedback, are likely to remain a standard component of trials. This study lays the groundwork for the development of a more integrated biomedical-behavioral approach to achieving high levels of adherence within HIV prevention trials. Ultimately, we envision being able to implement brief, easy to implement tools that identify attitudinal, partner-related or other contextual barriers likely to impede product adherence within trials. Once prospectively validated, the screening tool could be used to modify recruitment procedures, either screening out or more effectively addressing the concerns of potential participants whose propensity to adhere to product and trial requirements is low. Because such information would be collected prior to a participant’s randomization, it could complement biomarker adherence measures in determining the true efficacy of the product being tested. Finally, the periodic administration of monitoring tools to assess product-related beliefs and concerns could facilitate a more rapid and tailored intervention by clinic staff to better understand contexts of non-use, address product-related doubts, manage perceived side effects or ensure that participants do not misunderstand product-related benefits in ways that increase their risk of HIV.
